# Cardiac Rehabilitation Patients’ Accounts of Their Emotional Distress and Psychological Needs: A Qualitative Study

**DOI:** 10.1161/JAHA.118.011117

**Published:** 2019-06-01

**Authors:** Rebecca McPhillips, Peter Salmon, Adrian Wells, Peter Fisher

**Affiliations:** ^1^ School of Psychological Sciences Faculty of Biology, Medicine and Health Manchester Academic Health Science Centre University of Manchester United Kingdom; ^2^ Department of Research and Innovation Greater Manchester Mental Health Trust Manchester Academic Health Science Centre Manchester United Kingdom; ^3^ Division of Clinical Psychology Psychological Sciences University of Liverpool United Kingdom

**Keywords:** cardiovascular disease, mental health, quality and outcomes, Cardiovascular Disease, Mental Health, Quality and Outcomes

## Abstract

**Background:**

Psychological distress is prevalent among patients with cardiovascular disease and is linked to increased risk of future cardiac events. Cardiac rehabilitation (CR) is widely recommended for treating psychological distress but has been of limited benefit. This study aims to understand how distressed cardiac patients describe their emotional needs and the response of CR.

**Methods and Results:**

A qualitative descriptive study was conducted with 46 patients who screened positively for anxiety and/or depression. Semi‐structured interviews were held, and data were analyzed using a constant comparative approach. Patients described low mood and diverse concerns, including threat of another cardiac event, restrictions on their lives, and problems unrelated to their health. Patients described worrying constantly about these concerns, worrying about their worry, and feeling that worry was uncontrollable and harmful. Patients wanted to “get back to normal” but lacked any sense of how to achieve this and were reluctant to discuss their worries with CR staff. They hoped to recover over time, meanwhile seeking reassurance that they were responding “normally.” Patients were mostly dismissive of psychological techniques used in CR.

**Conclusions:**

These findings expose a conundrum. Distressed CR patients have diverse worries but do not generally want to discuss them, so they invest hopes for feeling better in time passing and reassurance. An intervention acceptable to CR patients would allow them to address diverse worries but without having to share the content of worries, would have “face validity,” and would address patients’ worry about worry. Metacognitive therapy is an intervention that might be suitable.

**Clinical Trial Registration:**

URL: https://www.clinicaltrials.gov. Unique identifier: NCT02420431.

**Clinical Perspective**

**What Is New?**

- Psychologically distressed cardiac rehabilitation (CR) patients worried about diverse concerns that went beyond the physical aspects of their illness and treatment, and they worried about their worrying.
- However, CR patients were reluctant to discuss their concerns with CR staff and were mostly dismissive of psychological techniques commonly used in CR.

**What Are the Clinical Implications?**

- Metacognitive therapy offers a potential response to distress in CR patients because it allows them to address their diverse concerns without having to disclose the content of their concerns.
- Metacognitive therapy also allows CR patients to address meta‐worry and has face validity as a skill that can be learned and practiced.

In 2015, there were an estimated 423 million cases of cardiovascular disease globally.^1^ Depression and anxiety are prevalent in cardiac patients; approximately 40% of myocardial infarction survivors report moderate to significant depressive symptoms,^2^, ^3^ and generalized anxiety disorder and panic disorder are more than twice as prevalent in coronary heart disease (CHD) patients than in the general population.^4^ Anxiety and depression in cardiac patients are strongly associated with poorer quality of life, increased risk of future cardiac events, increased mortality, and greater healthcare use and costs.^5^, ^6^, ^7^

Cardiac rehabilitation (CR) is widely seen as a vehicle for psychological intervention for cardiac patients.^8^, ^9^, ^10^, ^11^, ^12^, ^13^, ^14^, ^15^, ^16^, ^17^, ^18^ Guidelines for CR services in many countries recommend that CR include educational and psychological components in addition to physical exercise.^8^, ^9^, ^11^, ^12^, ^13^, ^14^, ^15^, ^16^, ^17^, ^18^ Recommended psychological components include stress management,^8^, ^9^, ^12^, ^16^, ^18^ relaxation training^16^, ^18^ and low‐level cognitive behavioral therapy (CBT) techniques, such as the worry decision tree, self‐instruction training, and cognitive challenging.^19^ Studies of CR delivery show that psychological components such as stress management and relaxation training are frequently included in CR programs delivered in high‐income countries. In New Zealand, stress management is provided in 94% of CR programs and relaxation training in 79%,^19^ and in England, 79% of CR programs include stress management sessions.^20^ However, poor attendance and adherence is well documented in CR.^21^, ^22^ In England, Wales, and Northern Ireland, where data on attendance at different components of CR are available, only 26% of CR patients receive relaxation training and only 18% receive group psychological talks that include stress management.^23^ For patients who do attend CR, there is limited effect on psychological distress. A meta‐analysis of CR for depression in CHD patients who attended found that CR had only a small effect,^24^ and other meta‐analyses of psychological components often included in CR, for example, relaxation training and problem solving, also show small effect sizes.^25^, ^26^ Guidance on formal treatment for psychological distress in cardiac patients recommends CBT.^15^, ^27^, ^28^ However, several meta‐analyses have examined the effects of CBT in CHD patients, and all report small effects compared with a range of comparison conditions.^24^, ^25^, ^29^, ^30^

Although quantitative evaluations of both routine CR and formal psychological interventions show limited benefit for cardiac patients, such evidence provides little information as to why this might be. Qualitative methods are increasingly used in evaluation of complex interventions, such as CR and CBT, to help understand quantitative evidence and to provide information on whether, and how, interventions meet patients’ needs.^31^ Consequently, UK Medical Research Council guidance advocates qualitative methods to explore patients’ own experiences of interventions and thus to help understand why expert‐designed psychological interventions sometimes fail.^32^, ^33^ Previous qualitative studies have examined cardiac patients’ perspectives on psychological distress. Patients experience a variety of negative emotions, including anxiety, depression, guilt, irritability, and anger, and attribute these to physical symptoms of CHD, comorbid health conditions, the limitations imposed by their disease, and uncertainty about the future. Depressive symptoms, in particular, impair self‐care and management of CHD.^34^, ^35^ However, qualitative studies have typically included unselected patients, that is, those who were not clinically distressed as well as those who were. Moreover, to our knowledge, only 1 qualitative study has explored depressed patients’ views on psychological care in CR^36^; only 6 patients in the usual‐CR control condition of that study were interviewed, and those patients recalled having minimal discussion of emotional needs. Therefore, the views and experiences of clinically distressed CR patients who have received standard CR remain largely unstudied. Knowing more about these patients’ perspectives could help understanding of the limited effects of CR on psychological distress. Taking a qualitative approach for this study, we recruited patients who were clinically anxious and/or depressed and interviewed them at various stages of CR, or shortly afterward, before they had received any other formal psychological intervention. We explored how patients described and understood their distress, how well they thought that current CR and routine care addressed their psychological needs, and how they viewed the potential role of formal psychological interventions.

## Methods

This study was conducted as part of the PATHWAY (Improving the effectiveness of psychological interventions for depression and anxiety in the cardiac rehabilitation PATHWAY using group‐based metacognitive therapy) trial (ethical approval from the UK Health Research Authority, North West Center of Research Ethics Committee [REC reference: 15/NW/0163]).^37^ PATHWAY is a randomized controlled trial (RCT) that compares usual CR alone with usual CR plus 6 sessions of group metacognitive therapy (MCT), delivered by trained cardiac staff or research nurses. The interview data collected for this study cannot be made freely available to other researchers because it contains potentially identifying information. Nevertheless, in accordance with recognized good practice for qualitative research, the analytic methods are described in detail in this article, and extensive data extracts are provided to substantiate and illustrate each element of the findings.^38^

### Participants

Patients referred to 3 CR services in England were screened by CR staff for eligibility for inclusion in PATHWAY. The delivery and content of CR at these 3 recruiting sites varied; each offered patients an initial one‐to‐one assessment, either face‐to‐face or by telephone, followed by group‐based programs in either hospital or community settings, with one site also offering one‐to‐one home‐based CR. Group‐based CR was delivered weekly, over 8 to 10 sessions, on a rolling basis, meaning that patients could “catch up” if they did not attend sessions consecutively. CR included gym‐based exercise sessions, and relaxation and stress management training were also offered during educational talks after exercise. Site C provided an additional, stand‐alone, 4‐week, stress management course delivered by an occupational therapist.

Inclusion criteria were scoring ≥8 on the depression and/or anxiety subscale of the Hospital Anxiety and Depression Scale (HADS),^39^ meeting CR eligibility criteria,^9^ being aged ≥18 years, and having sufficient competence in English to give informed consent. Patients were asked at the point of consent to PATHWAY whether they agreed to be contacted about qualitative research on their experiences of CR. Of the first 79 patients who consented to participate in the RCT, 77 agreed to be contacted; 60 of the 77 were purposively sampled to include women and men with different levels of distress, and 43 provided informed written consent to the qualitative study and were interviewed. Forty‐three patients who declined to take part in the RCT were also asked if they agreed to be contacted about qualitative research. We contacted all 15 who agreed, of whom 3 provided informed written consent and were interviewed. We interviewed patients at a range of stages of CR (Table 1), and patients randomized to the intervention arm of the RCT were interviewed before beginning MCT.

**Table 1:** Sample Characteristics

Identification Number	Sex	Age Range, y	Ethnicity	Highest Level of Educational Qualification	Employment Status	Relationship Status	Cardiac Condition	Comorbid Health Conditions	History of Treatment for Anxiety and/or Depression	HADS Scores	CR Sessions Attended Before Interview
P01	Female	45–54	Caribbean	GCSE or equivalent	In full‐time (paid) employment	Cohabiting	ACS (MI)	Hypertension; arthritis	No history of relevant medication; has received CBT previously	A=9D=3	2
P02	Male	45–54	White British	GCSE or equivalent	In full‐time (paid) employment	Married	ACS (MI)	Hypertension; high cholesterol	No history of relevant medication or psychological therapy	A=10D=3	3
P03	Female	65–74	White British	Vocational qualification	Retired	Divorced	Adult congenital heart disease	Hypertension; diabetes mellitus type 2; MS; COPD; arthritis; chronic fatigue; fibromyalgia; high cholesterol	Past medication for anxiety; current medication for depression; has received counseling previously	A=14D=10	4
P04	Male	45–54	White British	Diploma	In full‐time (paid) employment	Cohabiting	ACS (MI)	High cholesterol	Past medication for depression; has received psychological therapy previously (therapy type not disclosed)	A=8D=3	5
P05	Male	≥75	White British	Not collected	Not collected	Not collected	Not collected	Not collected	Not collected	Not collected	4
P06	Female	65–74	White British	None	Retired	Married	ACS (MI)	Hypertension; atherosclerosis; COPD; arthritis; incontinence	Current medication for depression; has never received psychological therapy	A=8D=8	3
P07	Male	55–64	White British	None	Unemployed	Separated	ACS (MI)	Hypertension; high cholesterol; COPD; arthritis; urethral trauma	No history of relevant medication or psychological therapy	A=10D=5	5
P08	Male	45–54	Asian	Diploma	Unemployed	Married	ACS (MI)	Hypertension; diabetes mellitus type 2; high cholesterol; atherosclerosis; COPD; hernia	No history of relevant medication or psychological therapy	A=9D=9	5
P09	Male	≥75	White British	None	Retired	Widowed	Stable heart failure	Hypertension; diabetes mellitus type 2; arthritis	No history of relevant medication or psychological therapy	A=2D=13	4
P10	Male	45–54	White British	None	Unable to work due to long‐term disability or ill health	Divorced	ACS; stable heart failure	Hypertension; high cholesterol; COPD	No history of relevant medication or psychological therapy	A=5D=12	4
P11	Male	55–64	White British	GCSE	Unable to work due to long‐term disability or ill health	Divorced	Adult congenital heart disease	Hypertension	No history of relevant medication or psychological therapy	A=10D=10	6
P12	Female	55–64	White British	Postgraduate degree	Retired	Single	ACS (MI)	COPD; IBS	Current medication for depression and anxiety; has received person‐centered therapy and mindfulness therapy previously	A=14D=11	5
P13	Male	45–54	Australian	Postgraduate degree	In full‐time (paid) employment	Separated	ACS (MI)	Hypertension; epilepsy	No history of relevant medication or psychological therapy	A=10D=8	5
P14	Male	45–54	White British	GCSE	Unemployed	Separated	ACS (MI)	COPD	No history of relevant medication or psychological therapy	A=7D=9	6
P15	Male	55–64	White British	None	In full‐time (paid) employment	Married	Adult congenital heart disease	Hypertension; COPD	No history of relevant medication or psychological therapy	A=15D=8	1
P16	Male	55–64	White British	None	Retired	Married	ACS (MI)	Hypertension; high cholesterol; IBS	Current medication for depression and anxiety; has received counseling previously	A=15D=11	5
P17	Male	55–64	White British	Degree	In full‐time (paid) employment	Married	Coronary heart disease	None	No history of relevant medication or psychological therapy	A=3D=8	2
P18	Male	45–54	Caribbean	A level	In part‐time (paid) employment	Single	ACS (MI)	Hypertension; diabetes mellitus type 2; high cholesterol; atherosclerosis; partially sighted	No history of relevant medication or psychological therapy	A=12D=5	4
P19	Male	35–44	Duel White British and New Zealand	Postgraduate degree	In full‐time (paid) employment	Cohabiting	Adult congenital heart disease	Hypertension	No history of relevant medication; has received counseling previously	A=8D=5	4
P20	Female	65–74	White British	None	Retired	Divorced	ACS (MI)	Hypertension/high blood pressure; diabetes mellitus type 2; atherosclerosis; arthritis; sciatica	Past medication for depression; current medication for anxiety; has never received psychological therapy	A=12D=11	4
P21	Female	55–64	White British	None	Retired	Married	ACS (MI)	Hypertension; COPD; arthritis; duodenal ulcer; brittle bone disease; glaucoma; blood clotting; cataracts	Past medication for depression; has received psychotherapy previously	A=9D=5	6
P22	Male	55–64	White British	Not Collected	Not collected	Not collected	Not collected	Not collected	Not collected	Not collected	Not collected
P23	Male	≥75	White British	None	Retired	Married	Stable angina	Hypertension; high cholesterol; arthritis; hernia; diverticulitis	No history of relevant medication or psychological therapy	A=14D=9	3
P24	Male	35–44	White British	None	Temporary sick leave	Single	ACS (MI)	High cholesterol; gout	No history of relevant medication or psychological therapy	A=11D=8	3
P25	Male	35–44	White British	Degree	Unable to work due to long‐term disability or ill health	Divorced	ACS (MI)	Blood clotting	No history of relevant medication or psychological therapy	A=15D=12	1
P26	Male	35–44	White British	GCSE or equivalent	Unable to work due to long‐term disability or ill health	Divorced	ACS (MI)	Hypertension; high cholesterol; back pain	Currently medication for depression; has received psychotherapy previously	A=9D=8	8
P27	Male	55–64	Asian British	None	Unable to work due to long‐term disability or ill health	Married	ACS (MI)	Diabetes mellitus type 2; high cholesterol; COPD; IBS or abdominal problems; kidney failure; cataracts	No history of relevant medication or psychological therapy	A=13D=15	6
P28	Female	35–44	White British	GCSE or equivalent	Temporary sick leave	Cohabiting	ACS (MI)	Arthritis	No history of relevant medication or psychological therapy	A=12D=11	3
P29	Male	45–54	White British	Vocational Qualification	Unable to work due to long‐term disability or ill health	Widowed	Adult congenital heart disease	COPD; arthritis	Past medication for depression and anxiety; has never received psychological therapy	A=11D=10	1
P30	Female	65–74	White British	None	Retired	Widowed	Adult congenital heart disease	None	No history of relevant medication; has received bereavement counseling previously	A=5D=10	10
P31	Male	≥75	White British	Vocational Qualification	Retired	Married	Atrial fibrillation	Hypertension; COPD	Current medication for depression and anxiety; has received counseling previously	A=10D=6	5
P32	Male	55–64	White British	None	Retired	Separated	Stable heart failure	IBS or abdominal problems; arthritis	Past medication for depression; has been treated by a mental health worker previously	A=11D=11	5
P33	Male	55–64	White British	Vocational qualification	In full‐time (paid) employment	Married	ACS (MI)	High cholesterol; sleep apnea	No history of relevant medication or psychological therapy	A=8D=6	5
P34	Male	55–64	White British	None	In full‐time (paid) employment	Married	ACS (MI)	Atherosclerosis	Past medications for depression; has been treated by a psychiatrist previously	A=12D=8	3
P35	Male	55–64	White British	A level	In full‐time (paid) employment	Divorced	ACS (MI)	COPD	No history of relevant medication or psychological therapy	A=8D=2	2
P36	Male	65–74	White British	Not collected	Not collected	Not collected	Not collected	Not collected	Not collected	Not collected	6
P37	Male	35–44	White British	Postgraduate degree	In full‐time (paid) employment	Single	ACS (MI)	None	Current medication for depression; has received web‐based CBT previously	A=9D=3	9
P38	Female	55–64	White British	Diploma	Unemployed	Single	Stable heart failure	Arthritis	Past medications for depression and anxiety; has received CBT previously	A=8D=3	9
P39	Female	≥75	White British	Diploma	Retired	Married	Stable heart failure	Hypertension; high cholesterol; arthritis; diverticilitis	Current medication for depression and anxiety; has received CBT and been treated by a psychiatrist previously	A=12D=16	1
P40	Female	65–74	White British	Vocational qualification	Retired	Divorced	ACS (MI)	Diabetes mellitus type 2	Current medication for depression; has received counseling previously	A=9D=8	7
P41	Male	45–54	White British	GCSE or equivalent	Unable to work due to long‐term disability or ill health	Single	ACS (MI)	IBS; Fabry disease; Crohn disease; renal failure	Past medication for depression and anxiety; has been treated by a psychologist previously	A=18D=19	3
P42	Male	≥75	White British	Diploma	Retired	Divorced	ACS (MI)	Hypertension; IBS; arthritis	Current medication for depression and anxiety; has received counseling previously	A=14D=13	6
P43	Male	55–64	White British	None	Unemployed	Single	ACS (MI)	None	No history of relevant medication or psychological therapy	A=9D=9	5
P44	Female	55–64	White British	Diploma	In part‐time (paid) employment	Divorced	ACS (MI)	Hypertension; diabetes mellitus type 2; arthritis; breast cancer	Current medication for anxiety; has received CBT and counseling previously	A=15D=3	2
P45	Male	45–54	White British	Vocational qualification	Unable to work due to long‐term disability or ill health	Separated	ACS (MI)	High cholesterol	No history of relevant medication or psychological therapy	A=16D=14	5
P46	Male	55–64	White British	Vocational qualification	In full‐time (paid) employment	Married	ACS (MI)	Breathing problems or COPD; IBS; arthritis	Past medication for depression; has received victim support counseling previously	A=9D=6	3

ACS indicates acute coronary syndrome; CR, cardiac rehabilitation; CBT, cognitive behavioral therapy; COPD, chronic obstructive pulmonary disease; GCSE, General Certificate of Secondary Education; HADS, hospital anxiety and depression scale (A, anxiety, D, depression); IBS, irritable bowel syndrome; MI, myocardial infarction; MS, multiple sclerosis.

### Procedure

R.M. interviewed patients privately in their homes (n=36), at her office (n=7), where patients received CR (n=2), or in a public cafe (n=1), as each patient requested. R.M. was the only author to meet with each patient, doing so for the first time at interview. Interviews, structured by an interview guide, were conversational, using open questions and prompts to facilitate patients’ talk and clarification questions to probe specific points (Table 2). Consequently, pace and sequencing depended on the patients. Patients were asked about emotional experiences since their cardiac event, experiences of CR, and whether and how they had been supported psychologically. To elicit their views about more formal psychological intervention (none had been offered), they were asked about having “somebody they could talk to individually or in a group to address emotional needs.” The interview guide was modified as the study proceeded to test and develop emerging ideas (Table 2). Interviews lasted 29 to 105 minutes (mean: 58 minutes).

**Table 2:** Interview Guides

Interview Guide 1: Used With Patients Who Took Part in the Study at the BeginningProbes Included in Brackets	Interview Guide 2: Used With Patients Who Took Part in the Study Toward The EndProbes Included in Brackets
Introduction to interviews Aims are for me to understand: What you think and how you feel about your experiences as a CR patient? How having a cardiac event might have affected you emotionally How you feel about the health care you have received so far	Introduction to interviews Aims are for me to understand: How having a cardiac event might have affected you emotionally How you feel about the health care you have received so far What you think about whether—and how—cardiac services should care for patients’ emotional health.
Part 1: Emotional experience since index event Ask about patient's cardiac event and entry into CR Ask about patient's emotional experience since cardiac event (How have you been feeling since your cardiac event? Have you felt sad, worried, frightened, irritable, angry?)	Part 1: Emotional experience since index event Ask about patient's emotional experience since cardiac event (How have you been feeling since your cardiac event? Have you felt sad, worried, frightened, irritable, angry?) Ask about patients’ concerns (Health? Family? Money? Work? The past? The future? Anything else?) Their frequency (how often do you think about these things? Just when someone asks you? Every week? Every day? Every hour? All the time?) Their duration (how long do you spend thinking about these things? A couple of minutes? Hours? All the time?) Triggers (someone asking how you are feeling? Physical sensations? Reading, watching or listening to certain things? Something else?)
Part 2: Interaction of emotional state with clinical care Ask if patient had any expectations about receiving help with the emotional impact of their cardiac event (Did you think that you would be given any help with how your cardiac event might affect you? What kind of thing did you expect? Information like leaflets? Someone to talk to among CR staff? To talk to the GP? To talk to someone else? Be referred to someone who could help? Anything else?) Ask what patient thinks about CR staff discussing patients’ emotional needs with them (Is this a good or bad idea? Why is that? Can you tell me a bit more about that?) Ask whether they have been offered, and accepted, any help for the emotional impact of cardiac events (eg, relaxation sessions, stress management, GP referral, CBT) and about what they think about any treatment that was offered If treatment was accepted and has been received, ask about this (Helpful or not?) If treatment was not accepted, ask why this was	Part 2: Interaction of emotional state with clinical care Ask if patient has spoken to anybody about the emotional impact of cardiac event (CR staff, other patients, family, friends, GP or other practitioner) Ask whether they have been offered, and accepted, any help for the emotional impact of cardiac events (eg, relaxation sessions, stress management, GP referral, CBT?) and about what they think about any treatment that was offered If treatment was accepted and has been received, ask about this (Helpful or not?) If treatment was not accepted, ask why this was Establish patient's views on discussing the emotional impact of cardiac event (eg, some experts have said that it would be useful if CR patients talked about their feelings—what do you think about that?) Explore whether patient would discuss his or her emotions with a member of the CR staff (e.g. If there were a member of staff whose job it was to talk to patients about how they feel emotionally, what would you do? Would you talk to them or not?) Explore whether patient believes distress after a cardiac event is normal (Do you think that the way that you have been feeling after your cardiac event is normal?)

CBT indicates cognitive behavioral therapy; CR, cardiac rehabilitation; GP, general practitioner.

Interviews were audio‐recorded, transcribed verbatim, and pseudonymized. We took a descriptive approach to the qualitative analysis, in which we explored and presented patients’ experiences and perspectives in words that kept as close as possible to their own accounts.^40^, ^41^ We followed a constant comparative approach that proceeded in parallel with interviews.^42^, ^43^ R.M. led the analysis. She and P.S. read and re‐read transcripts of the first 4 interviews, identified commonalities and contrasts in patients’ accounts, and began to develop a preliminary thematic framework relevant to the research questions. This approach facilitated a data‐driven synthesis of patients’ accounts.^40^ R.M. and P.S. then developed the thematic framework further, drawing in additional data from more transcripts, by organizing data into categories that described emerging elements of patients’ accounts that were relevant to the aims and that evolved as further transcripts were incorporated into the analysis.^44^ This framework was tested and refined in discussion with P.F., who also read the transcripts. During the final stages of analysis, the framework was tested by discussion among all authors, informed by re‐reading selected transcripts. During analysis we attended to possible sources of heterogeneity, including age, sex, study site, and previous experience of psychological interventions. We judged the developing analysis according to its “catalytic” and “theoretical” validity,^45^ whereby findings should have practical implications for the study population and should connect with broader theory. Analysis was inductive in that we aimed to present features of patients’ accounts that became salient through reading similar or contrasting ideas across patients’ accounts rather than because of their significance for a priori theories. We drew on theoretical frameworks after analysis was complete in considering the implications of the findings.

The analysis team encompassed a variety of theoretical and disciplinary backgrounds. R.M. has experience doing qualitative research on patient perspectives on physical health care. The other authors are clinical psychologists with expertise in physical health care: P.S. also has expertise in qualitative analysis of patients’ accounts of their psychological needs and experiences of health care; A.W. has contributed to the development of CBT in mental health and is the originator of MCT; P.F. has expertise in CBT and MCT and in providing psychological therapies in physical health contexts. Even in inductive research, researchers’ own perspectives inevitably influence the process and outcome of qualitative data analysis.^46^, ^47^ Therefore, to ensure reflexivity in drawing from the team's diverse perspectives, each member reflected throughout our discussions on how the different perspectives might be shaping his or her contribution to analysis.^48^ When competing interpretations were identified, we took a “disputational” approach whereby we tested them by rigorous debate based on the evidence provided by the data and informed by awareness of each member's perspective.

Data quotations were selected to illustrate and justify the analysis.^49^ Patients are identified by their participant numbers (eg, P01, P02, etc.); brackets indicate explanatory comments, and ellipses indicate omitted talk. As appropriate, we report the number of patients in whom we recognized specific findings. In the context of qualitative research, such “quantizing” is more concerned with internal than external validity.^50^ Therefore, we do not seek to make generalizations based on these quantities; rather, we seek to demonstrate how broadly our analysis is supported by the data.

## Results

### Patients Described Diverse Concerns

Consistent with the HADS inclusion criterion, patients described feeling unhappy since their cardiac crisis: “I feel down… I get upset” [P45]; “I have this sort of cloud that comes down… I could burst into tears” [P40]; “It's as if my whole mental system… is just closing down… I feel depressed” [P31]. A striking feature of patients’ accounts was the extent of their worrying**,** which could take “80% of [their] waking time” [P26] and “it could be 24 hours a day… the negative thoughts could just be constant” [P37]. Patients worried and ruminated about a number of concerns, including ones that were unrelated to their health and that predated their cardiac event. Our analysis centered on concerns that were salient to patients and on patients’ feelings of helplessness in managing worry about them. We did not see differences between accounts of men and women or patients from different centers. Where other systematic sources of heterogeneity arose according to age or previous experience of psychological interventions, we report them.

#### Another cardiac event

Nearly all described feeling “scared” [P08] about another cardiac event: “It's going to happen again… That's in your mind all the time, it's going to happen again” [P24]. Although patients described finding it “scary suddenly realizing that, you know, you could die any time” [P12], this fear often drew in other concerns about the future, as P22 illustrated:

“I've done damage to my heart… you've been wounded and you think, ‘well, actually, what's my chances now of living another 20 years?’ At my age, if I got to 82, I'd be very happy… I have a grandchild who I'd like to see grow up… I'm not ready to give it up, you might say” [P22].

Worrying about a further cardiac event was linked to being alert to symptoms, including “every little twinge” [P43], even if patients would previously have assumed benign causes:

“Your pectoral muscles here, if you pulled one of them, you would think it was heart attack related. Everything is heart attack related, once you've had a heart attack” [P28].

Patients also worried about their own behavior, for example, asking themselves, “am I doing too much?” [P24], or “have I taken the medication?” [P12]. P40 was concerned about whether, by “overdoing it,” she would “undo” the benefits of her coronary bypass surgery. Patients ruminated on the events leading up to their cardiac event, too. For example, P46 continued to “analyze” the day he had a heart attack so that he “would know what to do next time.”

#### Life becoming restricted

Patients’ concerns extended beyond fearing another cardiac event. They ruminated about things they used to do, particularly involving physical activity, as having been “taken away” [P13] by their illness. P27 described how “you do get sad sometimes… thinking like a few months back you were fit, walking about… suddenly the attack came and you just change your life altogether.” Several patients described ruminating about lost hopes, for example, P17 described:

“You work hard all your life and you're approaching your time and you're thinking… in retirement, you're in good health, you won't be messing around with operations and things that are going to prevent you doing the things that you want to do when you've got a bit more time, so it was disappointing that the plans you had in the back of your mind might not come to fruition” [P17].

Many described feeling “restricted” by clinically recommended lifestyle changes, particularly around smoking and alcohol.

#### Self‐criticism

Patients consistently described recurrent thoughts and worries that denigrated themselves, for example, feeling “stupid for feeling like this” [P30]; feeling that they “can't be bothered” [P41]; or that they had had become “short tempered” [P25], “snapping too quick” [P15], ”pathetic” [P39], “old… and useless” [P30]. Several contrasted “who you are and who you were” [P13], explaining that they felt like “a different person” [P38] since their cardiac event. P15 explained, “I get ever so upset now about absolutely nothing… I'd never get upset that quick… before I had it [valve replacement] done.”

#### Concerns unrelated to cardiac health

Almost every patient described concerns that were not directly related to their cardiac history. Several reported dwelling on regrets about their previous life: “you move away from your friends and you think, ‘it wasn't worth it’” [P17]; “my childhood hasn't been brilliant, my relationship with my dad is not very good” [P41]. Almost all patients also described current worries unrelated to their health. A few were concerned about lack of money: “it's mainly money… I ain't got no money” [P10]. Many, however, described concerns about close relationships: “my wife, she left… the children are upset about it, that's what bothers me the most, it's quite difficult to deal with” [P45]; “my daughter is an alcoholic… last year she had a massive bleed… they didn't think she would survive… [it is] a huge worry” [P40].

#### Concerns that worry might be harmful

Many patients worried about worry. They worried that “stress has played a big part in me being ill” [P25]. For example, P44, who described feeling “anxious most of the time,” blamed herself for her heart attack, thinking that “if I was less anxious and less stressed I probably wouldn't have had it.” Patients worried that their current worry and distress was a danger for the future:

“You worry about worry, so that worries you… when I was anxious before, nothing ever happened to me… if I'm anxious now, I don't know if something will happen to me” [P11]; “If you worry about things, you're going to self‐stress, and that is one of the indicators for more heart problems” [P35].

### Worrying Felt Uncontrollable

When patients described their worrying, they consistently referred to having “no control” [P06] over it, explaining, for example, that “I can't stop worrying about the kids” [P43]. P17 feared no longer being able to retire because, apart from working, he saw no other way to stop worrying: “I've got to keep working… your mind would be more active, you can't always be thinking, ‘what can I do now?’” Patients linked the excessive time they spent worrying to their feeling that they had no way to stop the worry: “[I worry] all the time really… I could be driving down the motorway… but my mind is ticking away with things all the time… you get home and you just don't seem to be able to switch off” [P41].

### Patients Wished to “Get Back to Normal” and Sought Reassurance From Peers and Staff That They Would

Patients wanted to get back to “how it was before” [P46], as P22 explained: “the goal that I've got in my head is to actually just forget about the fact that I have had a heart attack… so that I don't wake up in the morning thinking, ‘oh God, you've had a heart attack.’… it would just bring me back to a sense of normality.” However, patients did not know how they could achieve this. Rather, they hoped that they would recover with time. For example, P24 hoped that he could eventually “forget” about feeling tearful while anticipating that this was “going to take a long time.” P23 explained that “it's hard to pull myself out” of a low mood and that he was simply “hoping it'll fade away.”

Patients sought reassurance that their physical symptoms and emotional difficulties were “normal,” that “you're supposed to be up and down” [P46]. When P40 was asked what emotional support CR patients needed, she responded “that it's normal, to feel like this… or be warned that there is a possibility that you could… have these worries and anxiety.”

The need for reassurance that their experiences were normal explained why almost all patients wanted to exchange accounts with others who had been through the same experiences: “it'd be nice to hear what people have got to say… see if I am perfectly normal and everybody feels the way I feel or if I'm the odd one out… you want to know if… what's going to happen to me probably happens to everybody” [P46]. Patients who had been able to talk with others had found it reassuring:

“I was saying [to other patients] I've got this pain… and somebody said, ‘Oh well, I've had that.’ And I said, ‘oh, have you?’ You know, it's… quite reassuring” [P12].

The most valuable exchanges that patients envisaged were with others who were “three years on from you… then it's going to be a great help, because you can think, ‘hang on, there's nothing going to happen to me because he's been doing work, he's been cycling, swimming for three years and he's right as rain’” [P11]. However, five patients, all of whom were <55 years old, described feeling too “embarrassed” for this kind of disclosure, specifically stating that they “wouldn't like to be in a room full of oldish men and then open up” [P24].

A few patients described also gaining reassurance from staff, as P22 illustrated: “I'm a bit more positive now… I think it's getting confirmation and affirmation from various doctors… that what I am experiencing now is normal for my situation.” Reassurance generally proved transient, however. P43 described how, when CR staff reassured him that “everything's fine… that puts my mind at rest”; but when asked how long the benefit lasted, he replied, “mostly that day and sometimes it's the next day.” Moreover, most patients explained that they did not receive the reassurance they sought from staff. Some complained that services did not make staff accessible when patients needed support, for example, because CR starts “too late,” or “nobody had been in touch” [P15] following discharge, so patients felt “on their own” [P12]. P34 explained, “It doesn't seem right after someone has had a heart event… I need to talk to someone… and they just didn't want to know… you come out of hospital and there is nothing.” Mostly, however, patients described staff as “busy” [P20], having “other patients to deal with” [P07], or being “there to deal with the physical… just for exercises” [P12]. When prompted further, however, patients consistently linked these attributions to their own discomfort at discussing “personal” or “private” matters with staff. Discomfort was compounded because “they [nurses] kept changing… you'd have then the next week a different [nurse] who you didn't know” [P37]. For many patients, the cardiac care environment felt inimical to disclosure. For example, P28 did not “want to go and have a work‐out in the gym once you've poured your heart out to somebody,” and said she would not disclose her worries even if a member of staff were provided specifically for emotional support because “it's not the right environment.” Several patients contrasted the discomfort they would feel disclosing to cardiac and CR staff with the ease of talking to other patients:

“I can talk to people [other patients] because they're the people who have had exactly the same problem as me… I think I am a bit of a private person to be honest… If I am talking to the nurses… obviously they can't, how can I say, sit there for say *X* amount of minutes or hours or whatever talking to me one‐on‐one because they've got other patients to deal with, and this is what I think about” [P07].

### Patients Were Generally Negative About What They Understood as Psychological Intervention

Twenty‐six patients recalled being offered relaxation and stress management as part of CR. Eight had declined, thinking them unhelpful: “when I go for exercise, I like to do exercise… I don't want to get other things involved” [P27]. Of 15 patients who had already attended, only 5 found them helpful, for example, to “manage stress” [P37]; “that's [progressive muscle relaxation] my way of getting to sleep” [P31]. The others who attended dismissed the techniques as superficial: “the problem with relaxation is… [it] really isn't exercise for the mind” [P06]. Some doubted being able to implement the techniques: “I just can't do it [guided relaxation] on a regular basis… I don't think I'd be able to do it” [P10]. Similarly, P04 found that a talk on stress “makes sense… but… I could tell myself all day, ‘it's not my fault, there's nothing I can do about it, forget about it.’… my mind won't let me do that” [P04]. Two suggested that relaxation was a skill that should be practiced “after every one [CR session]… try and get it ingrained, a behavioral change” [P19].

As well as asking about the psychological components of CR that patients had been offered, we prompted patients about being able to address emotional needs with a dedicated member of staff individually or in a group. Patients generally assumed this would mean talking about “what they went through, what their feelings were” [P02]. Most initially agreed that this kind of help would be valuable, but further prompting showed most patients’ assents as “politeness,” because they went on to indicate that they saw this kind of talk as not “belonging” in the cardiac context or as an opportunity they would decline. For example, P25 responded that “I think it's a really good idea… someone… might not need to go in a gym, they might need to talk to someone,” but later observed that “you don't want to open up really to a stranger” [P25]. Fifteen patients, however, explained that they thought having psychological help that involved talking about their emotional problems would be useful. For example, these patients described needing “somebody to help me look at myself” [P44] or wanting “to direct my thoughts into better ways than I am doing at the moment” [P42]. Most (n=10) of these patients were among the 18 who told the interviewer of previous experience of intervention for psychological distress (although we did not explicitly ask about this).

## Discussion

The CR patients in this study, who all screened positively for anxiety and/or depression, described distress that encompassed low mood and diverse concerns. For many patients, worry about these concerns felt overwhelming.

Patients had diverse concerns related to their cardiac disease, including fear of dying or of having their life restricted by disability or continuing treatment, and their sense of loss because of the contrast between their current and former senses of self. Most also had serious concerns that were unrelated to cardiac disease or that even predated it. Although many of these concerns reflect evidence from previous studies,^34^, ^35^ our findings go beyond describing the range of cardiac patients’ concerns to highlight the intractability of worry for distressed patients; they worried about worry itself and believed that worrying is uncontrollable and harmful. Whereas the concerns and beliefs CR patients have about worry have not been described previously, they are central to the metacognitive model 51 of psychological distress and are potentially helpful in understanding how CR patients’ distress might be better addressed, given that patients experienced psychological aspects of CR as too superficial to help them.

Patients wanted to “get back to normal” and stop worrying. Feeling that they lacked any way to help achieve this other than waiting for the time to pass, they sought reassurance from staff and peers that they were responding “normally.” However, reassurance was generally transient, consistent with previous findings that reassurance has little benefit for patients with cardiac symptoms.^52^, ^53^ A new and potentially important element of our findings was that, despite wanting reassurance, most patients were reluctant to talk about their worries in the context of CR unless they had been previously socialized into psychological interventions. Furthermore, despite being so troubled by worry, most were dismissive of stress management and guided relaxation techniques offered in the context of CR. These techniques seemed superficial and difficult to apply in real‐life and needed more practice than CR provided.

Consequently, these results expose a conundrum: distressed CR patients describe being trapped in a cycle of negative thinking but see no way of relieving this, apart from passively waiting for time to pass and seeking reassurance that the distress and worry they experience is normal. They see existing psychological components of CR—stress management and relaxation training—as superficial and unhelpful, but most patients do not want to engage with psychological intervention that would mean disclosing “private” worries or concerns.

Our findings resonate with previous research that found patients associate CR predominantly with physical exercise.^21^ This perception might be a barrier to using CR as a vehicle for psychological intervention. However, our findings point to properties that might enhance the acceptability of psychological intervention to distressed CR patients. First, an intervention would address the diverse worries that such patients can have, extending from fears of dying after another cardiac event to concerns that long predate their cardiac history. An intervention would also address patients’ metaworry and metacognitive beliefs that worry is uncontrollable and harmful. Second, rather than be a superficial addition to CR, an intervention would have “face validity” as a skill that patients can learn and practice to “exercise the mind.” Third, it would allow patients to keep the content of their worries private if they wished. Existing psychological approaches for distressed CR patients do not compare well with these criteria. In particular, our findings suggest that stress management and guided relaxation techniques as part of CR lack face validity as skills that can plausibly counter the extent and diversity of distressed patients’ worries. As a content‐focused therapy, CBT is inconsistent with patients’ wish not to disclose concerns in the cardiac context. Given the limited benefits associated with CBT and other psychological approaches to managing distress in CR patients, an intervention is needed that is not only acceptable to CR patients but also has the potential to be effective in this population. The accounts of distressed patients in this study indicate that a treatment that enables the individual to learn to control worry while reducing the tendency to worry about worry might be an effective approach.

MCT offers a potential solution for cardiac patients because it is aimed at bringing worry and rumination under control without the need to examine worry content.^51^ This is achieved by targeting metacognitive processes—metaworry and metacognitive beliefs—using techniques including attention training and detached mindfulness, which can be practiced.^51^ The beliefs of patients in this study that worry is uncontrollable and harmful are examples of the metacognitive beliefs that figure prominently in MCT.^51^ Most patients in this study placed value on interacting with other patients, a finding that resonates with previous research; therefore, group‐based therapy may be of particular benefit.^36^ Furthermore, because MCT is a process‐focused therapy and focuses on the metacognitive domain of the patient, it allows patients to keep the content of worry private if they so wish.^51^ MCT is transdiagnostic; therefore, a wide range of concerns, including any that preceded cardiac illness, can be addressed by this treatment, and meta‐analysis has shown that MCT is an effective treatment for both depression and generalized anxiety disorder.^54^ MCT might thus help CR patients, including those whose distress predates their cardiac event and whose concerns are broader than their physical health. Further support for the relevance of MCT for patients whose psychological distress is comorbid with physical illness comes from evidence of an association between metacognitive beliefs and psychological distress in several clinical contexts including cancer 55, 56 diabetes mellitus,^57^ epilepsy,^58^, ^59^ fibromyalgia,^60^ chronic fatigue syndrome 61, 62 and Parkinson's disease.^63^, ^64^, ^65^

## Limitations

Because this study is qualitative, the findings cannot necessarily be generalized. In addition, nonattendance at and nonadherence to CR are well documented,^21^, ^22^ and the views of patients who did not attend CR are not represented in this study.

Qualitative interview data collection and analysis are necessarily shaped by the researchers’ own perspectives.^45^, ^46^ This study was conducted in the context of an RCT of MCT, and the authors’ knowledge of the metacognitive model may have sensitized them to aspects of patients’ accounts, for example, worry about worry, that would not otherwise have been noticed. However, although 2 authors (A.W. and P.F.) are advocates of MCT, the other authors (R.M. and P.S.) are not; rather, their experience of using qualitative methods to provide patients’ perspectives with a voice and of challenging idealized theories meant that the research team encompassed a variety of theoretical perspectives. The disputational approach taken toward analysis ensured that the analysis was scrupulously tested and that it remained inductive while allowing its interpretation in the context of areas of theory that have the potential to improve patient experiences and outcomes.

## Conclusions

This study explores the perspectives of CR patients who are clinically anxious and depressed and is the first to highlight the intractability of worry for these patients, their worry about worry itself, and their beliefs that worry was uncontrollable and harmful. In the context of research that has found that CR and formal psychological interventions have been of limited benefit to patients, our findings help explain why the psychological needs of CR patients are not well met by current approaches and indicate characteristics that new approaches would need to be acceptable to CR patients.

## Sources of Funding

This research is funded by the National Institute for Health Research (NIHR) under its Program Grants for Applied Research Scheme (RP‐PG‐1211 20011). The views and opinions expressed are those of the authors and do not necessarily reflect those of the NIHR, the National Health Service, or the UK Department of Health and Social Care.

## Disclosures

Adrian Wells is a codirector of the MCT Institute and chief investigator on grant RP‐PG‐1211‐20011. The remaining authors have no disclosures to report.
